# Exploring virus release as a bottleneck for the spread of influenza A virus infection in vitro and the implications for antiviral therapy with neuraminidase inhibitors

**DOI:** 10.1371/journal.pone.0183621

**Published:** 2017-08-24

**Authors:** Laura E. Liao, Szymon Kowal, Daniel A. Cardenas, Catherine A. A. Beauchemin

**Affiliations:** 1 Department of Physics, Ryerson University, Toronto, ON, Canada; 2 Interdisciplinary Theoretical and Mathematical Sciences (iTHES, iTHEMS) research group at RIKEN, Wako, Japan; Tokyo Institute of Technology, JAPAN

## Abstract

Mathematical models (MMs) have been used to study the kinetics of influenza A virus infections under antiviral therapy, and to characterize the efficacy of antivirals such as neuraminidase inhibitors (NAIs). NAIs prevent viral neuraminidase from cleaving sialic acid receptors that bind virus progeny to the surface of infected cells, thereby inhibiting their release, suppressing infection spread. When used to study treatment with NAIs, MMs represent viral release implicitly as part of viral replication. Consequently, NAIs in such MMs do not act specifically and exclusively on virus release. We compared a MM with an explicit representation of viral release (i.e., distinct from virus production) to a simple MM without explicit release, and investigated whether parameter estimation and the estimation of NAI efficacy were affected by the use of a simple MM. Since the release rate of influenza A virus is not well-known, a broad range of release rates were considered. If the virus release rate is greater than ∼0.1 h^−1^, the simple MM provides accurate estimates of infection parameters, but underestimates NAI efficacy, which could lead to underdosing and the emergence of NAI resistance. In contrast, when release is slower than ∼0.1 h^−1^, the simple MM accurately estimates NAI efficacy, but it can significantly overestimate the infectious lifespan (i.e., the time a cell remains infectious and producing free virus), and it will significantly underestimate the total virus yield and thus the likelihood of resistance emergence. We discuss the properties of, and a possible lower bound for, the influenza A virus release rate.

## Introduction

There are two main classes of antiviral drugs available for the treatment of influenza A virus infection: adamantanes, such as amantadine and rimantadine, and neuraminidase inhibitors (NAIs), such as oseltamivir, zanamivir, laninamivir, and peramivir. In 2005–2006, however, resistance to adamantanes dramatically increased [1], and the currently circulating influenza A/H3N2 strains are adamantane-resistant. In light of this, the World Health Organization primarily recommends NAIs such as oseltamivir and zanamivir for antiviral therapy against currently circulating strains of influenza A virus [2]. As a part of pandemic preparedness planning, oseltamivir has been stockpiled worldwide. However, even oseltamivir-resistant A/H1N1 strains have emerged and circulated [3]. Recent focus has turned to the development of new antivirals that inhibit viral polymerase (e.g., favipiravir), though these have yet to be approved in most countries, leaving NAIs as the leading antiviral approved for the treatment of influenza A virus infections.

NAIs reduce the spread of influenza A virus to uninfected cells by blocking the release of progeny virus produced by infected cells. At this late step in the viral replication cycle, mature virions protrude and pinch off from the apical surface of the infected cell, co-opting the cell’s plasma membrane as their own envelope, but can remain affixed atop the cell surface. Both the virion and the cell surface, which is destined to become the virion’s outer surface, are studded with the viral proteins neuraminidase (NA) and hemagglutinin (HA), as well as the cell’s sialic acid receptors. Throughout the duration of the infection, increasing amounts of NA are expressed on the cell surface, which cleave sialic acid receptors. As the density of sialic acid receptors declines, newly budded virions are less likely to remain cell-bound due to the formation of virus-cell attachments when HA binds to the sialic acid receptors upon exit. We will refer to the transition from cell-associated, bound virus into free virus that is facilitated by NA cleavage of sialic acid cell receptors as “virus release”, though other modes of virus release might exist [4–6].

**Fig 1:**
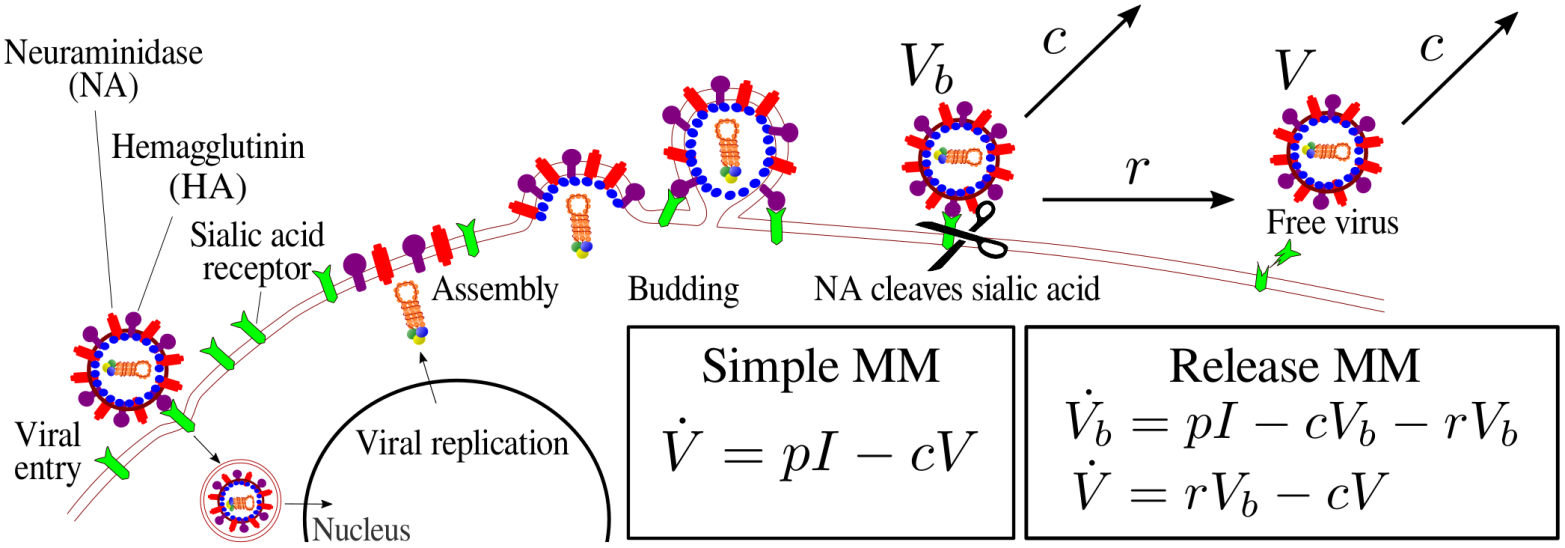
Modelling influenza A virus infection with and without explicit release. In an influenza A virus infection, the virion gains entry into the cell when hemagglutinin (HA) proteins on the surface of virions bind to sialic acid receptors on the surface of the target cell. As viral replication gets underway, increasing amounts of viral proteins such as HA and neuraminidase (NA) are expressed on the cell surface. Throughout the infection, the density of sialic acid receptors declines as NA cleaves them. After viral replication takes place in the nucleus, the viral RNA progeny is transported to the cell membrane for virus assembly and budding. Some progeny virions will be released as free virus (*V*), while others remain bound (*V*_b_) to the cell surface upon exiting the cell when HA on the surface of budded virion binds to sialic acid on the cell. The simple MM without an explicit term for viral release encapsulates these later processes implicitly as a part of the parameter quantifying free virus production (*p*) by infectious cells (*I*). The release MM has an explicit term for viral release at a rate *r*, which occurs after bound virus is produced at a rate *p* onto the surface of the cell. In both MMs, all virions, bound (*V*_b_) or free (*V*), lose infectivity at rate *c*.

As reviewed in [7], a simple MM has provided insight into influenza A virus infection kinetics in both in vitro and in vivo settings. The simple MM has been used to study NAI therapy in humans that were infected with human strains [8] or avian strains of influenza A virus [9], and the MM has been extended to include an immune response [10, 11]. Although the simple MM has been used to study the inhibition of virus release by NAIs, it does not possess an explicit representation of virus release. In the simple MM, virus release is implicitly represented as part of virus replication which encompasses many processes, shown in Fig 1, such as viral transcription and translation, up to later events such as bud initiation, bud growth and closure, and finally virus release. Consequently, when NAIs are incorporated into the simple MM, they act on these combined processes instead of acting specifically and exclusively on virus release.

If virus release plays a significant role in the unfolding infection kinetics, the use of a MM without explicit virus release, like the simple MM, could affect the estimation of infection parameters, with broad implications. For instance, an estimate of the virus production rate can shape our perspective on the likelihood of the emergence of drug resistance [12–14]. In a scenario where rapid virus production (to be understood here as genome production/replication) is undermined by slow virus release from an infected cell, only the overall slow rate of appearance of progeny virus in the supernatant would be observed when the infectious virus yield is collected in vitro. If the simple MM, where the virus release rate is implicitly represented in the term for virus production, was used to fit these data, the estimated virus production term (representing genome production and release) would be an underestimation of the true rate (high genome production, low rate of release). Since the chance of generating drug-resistant mutants with a single amino acid substitution is predicted to increase as more virus genomes are produced [15], underestimation of the virus production rate will underestimate the likelihood of drug resistance emergence and the number of drugs, or efficacy, required to prevent resistance [16, 17].

The importance of quantifying the infection parameters from time course data of an in vitro infection with a MM has been discussed [18]. This approach allows for the quantitative comparison of differences between virus strains, for example, in the virus production rate, cell lifespan, or basic reproductive number, which are indicators of virulence, cytopathicity, and fitness. These quantities, even if determined only from in vitro infections, can inform and to an extent predict epidemiological outcomes such as pathogenicity and transmissibility [19].

The challenge to including virus release explicitly in a MM is that the influenza A virus release rate is not well-known. From a mathematical modelling standpoint, the addition of an explicit release rate to the simple MM will result in overparameterization, such that at least some parameters would suffer with identifiability issues. To overcome this challenge, our study considers a range of possible values of the release rate of influenza A virus, and compares the simple MM against a variation of it that includes an explicit term for virus release (Fig 1). We identify the critical release rate below which virus release begins to play a significant role in determining the levels of free (i.e., no longer bound to the cell) infectious virus. We show how neglecting to account for virus release with an explicit release term affects parameter estimation, as well as estimates of NAI efficacy.

## Results

### Considering an explicit release rate and the impact on viral kinetics

We compared a simple MM (without explicit virus release) to a MM with explicit virus release (hereafter, referred to as the release MM). As described in the Methods, the release MM is an augmented version of the simple MM with one additional parameter, the virus release rate *r*. Due to this small modification, the MMs have different views of virus infection, and some parameters in the two MMs require a slightly different interpretation. For example, in the simple MM, the eclipse length, *τ*_*E*_, represents the time it takes for an infected cell to replicate virus *and* release it. In contrast, in the release MM, the eclipse length only represents the time it takes for the infected cell to replicate virus, but the time to viral release is handled separately with parameter *r*. The interpretation of the virus production rate, *p*, in either MM also differs: whereas in the release MM, a fraction of the virions produced will never be released if the release rate is low, in the simple MM, all produced virions are released. Thus, in the simple MM, the effects of virus release can be absorbed by either or both the virus production rate and eclipse length.

Another difference from the simple MM, is that the release MM explicitly describes cell-associated, bound infectious virus (*V*_b_) being released at rate *r* as free infectious virus (*V*) into the medium. When we refer to bound virus, we mean infectious virions that are still attached to the virion-producing cell, i.e., cell-associated infectious virus. Experimentally, bound virus is not measurable in a typical cell culture experiment where only the supernatant is removed and titrated for infectious virus. On the other hand, free virus here refers to infectious virus that has successfully been released from virus-producing cells into the supernatant, and corresponds to the population of virus that is typically quantified in an experiment.

**Fig 2:**
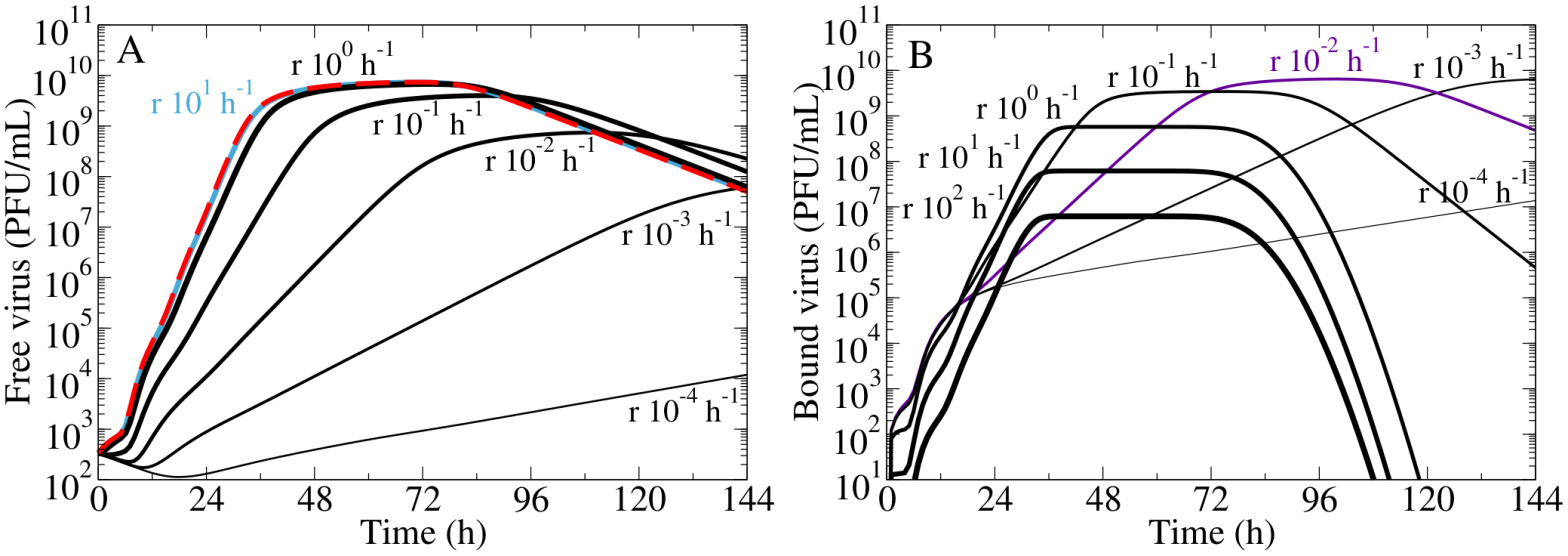
The role of explicit viral release on free and bound virus kinetics. (A) The concentration of free virus titer from an in vitro infection experiment with the 2009 pandemic influenza A (H1N1) virus strain [20] was simulated in the simple MM (red), which serves as our baseline simulation. The baseline is compared to the free virus titer simulated in the release MM (black) where the release rate, *r*, is varied from 10^−4^ h^−1^ to 10^−2^ h^−1^, and all other parameters are kept at their base values. Note that when *r* ≥ 100 h^−1^, the release MM reduces to the simple MM. For low release rates, the free virus is suppressed compared to the baseline (black vs red), but is unaffected when *r* is greater than a critical value of *r*_*f*_ = 3 h^−1^−5 h^−1^ (teal). (B) The corresponding concentration of bound virus in the release MM is shown (black), where the bound virus titer peak is maximal at another critical value of the release rate, *r*_*b*_ = 3 × 10^−3^ h^−1^ (purple).

We investigated the impact of the release MM on the kinetics of free and bound virus by comparing it to that predicted by the simple MM. In Fig 2A, a baseline simulation of the free virus kinetics predicted by the simple MM is shown for the case of a virus infection in vitro where the infectious virus released into the supernatant was quantified by plaque forming assay [20]. The release MM was used to simulate the same virus infection, with the same parameter values used with the simple MM, but the release rate was varied since the value of the influenza A virus release rate is unknown.

Compared to the simple MM simulation, the growth of free virus in the release MM was only suppressed when the virus release rate fell below some critical value. We call this value the critical free virus release rate, *r*_*f*_, and determined that it ranges between 3 h^−1^–5 h^−1^ (see S1 Supporting Information for details of this estimation). If the virus release rate is below this critical value, virus release is slow and inefficient and plays a significant role in suppressing free virus yield.

The release MM also predicts the effect of virus release on the kinetics of bound virus. In Fig 2B, the corresponding bound virus titer predicted for various release rates is shown. Note that this quantity is zero in the simple MM which assumes that all produced virus is released. Increasing the release rate from 10^−4^ h^−1^ to 10^−2^ h^−1^ increased the bound virus peak, but a release rate greater than 10^−2^ h^−1^ diminished the bound virus peak. There appears to be a value of the release rate that maximizes the bound virus titer peak, which we call the critical bound virus release rate, *r*_*b*_. We determined that *r*_*b*_ was approximately 3 × 10^−3^ h^−1^, though its value depends on other infection parameters (see S1 Supporting Information). When the release rate is high, virions are released as soon as they are produced, and the amount of bound virus is negligible. As the release rate is lowered, towards *r*_*b*_, produced virus remains attached longer and the concentration of bound virus increases. However, as the release rate is lowered even further, below *r*_*b*_, virions are trapped on the cell surface for so long that some will lose infectivity (their ability to infect other cells) before they are released, resulting in a decrease in the bound virus titer. A balance between these two processes results in the largest bound virus titer peak value.

Our results show that virus release can act as a kinetic bottleneck to significantly suppress free virus growth if the true rate of influenza A virus release is less than the critical free virus release rate, *r*_*f*_. In such a case where virus release plays a significant role in virus infections, release must be explicitly accounted for in the MM.

### The effect of explicit virus release on parameter estimates

Most MMs to date have represented the virus release as an implicit part of virus replication. Here, we are interested in how parameter estimation using the simple MM compares to that using the release MM over a wide range of possible release rates.

**Fig 3:**
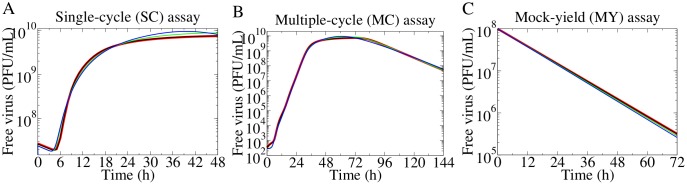
Simultaneous fits of the release MM to the simulated free virus titer of the SC, MC, MY assays. The release MM was simultaneously fitted to the free virus titer from the (A) SC, (B) MC, and (C) MY assays simulated using the simple MM (red; using base parameters). The production rate, infection rate, rate of loss of virion infectivity, eclipse length, and infectious lifespan were fitted. Fitted curves are shown for a high (*r* = 1 h^−1^, black), an intermediate (*r* = 0.1 h^−1^, green), and a low (*r* = 0.01 h^−1^, blue) release rate.

We have previously shown that a full suite of in vitro experiments—single-cycle (SC), multiple-cycle (MC), mock-yield (MY)—is needed to extract and identify the infection parameters (e.g., virus production rate, infection rate, rate of loss of virion infectivity, eclipse length, infectious lifespan) in the simple MM [20–22]. Briefly, the MC and SC assays are in vitro infections where a monolayer of confluent, uninfected cells are inoculated with a low or high concentration of virus, respectively. The initial conditions of infection are expressed in terms of multiplicity of infection (MOI), i.e., the number of cells infected by the initial inoculum over the total number of cells. Herein, the simulated MC assay was carried out at a MOI of 5 × 10^−5^ infectious virus/cell, while the SC assay was performed at a MOI of 4 infectious virus/cell. In the MY assay, the virus inoculum is incubated in the absence of cells and sampled at multiple times, exhibiting the rate of loss of infectious virions (a virus stability analysis). Fig 3 shows the free virus kinetics from these assays, as simulated by the simple MM using the base parameters reported in the Methods (Table 1), and by the release MM.

**Table 1:** Base parameter values for infection with an influenza A (H1N1) pandemic strain [20].

Parameter, *symbol*	Base value
Virus production rate, *p*	6.21 × 10^8^ (PFU/mL) ⋅ h^−1^
Virus infection rate, *β*	1.18 × 10^−8^ (PFU/mL)^−1^ ⋅ h^−1^
Rate of loss of infectious virus, *c*	7.98 × 10^−2^ h^−1^
Infected cell eclipse length, *τ*_*E*_	6.63 h
Infected cell infectious cell lifespan, *τ*_*I*_	48.9 h
Number of eclipse compartments, *n*_*E*_	30
Number of infectious compartments, *n*_*I*_	100

For the release MM, the release rate was fixed, and the remaining five MM parameters were fitted using the simulated free virus titer from the simple MM as the data to fit (red). A set of fitted parameters was obtained for each value of the release rate explored. The release MM can reproduce the simple MM’s infection kinetics with a unique set of infection parameters, for any given value of the release rate. This shows how the parameter estimates shift under different assumptions of the release rate.

**Fig 4:**
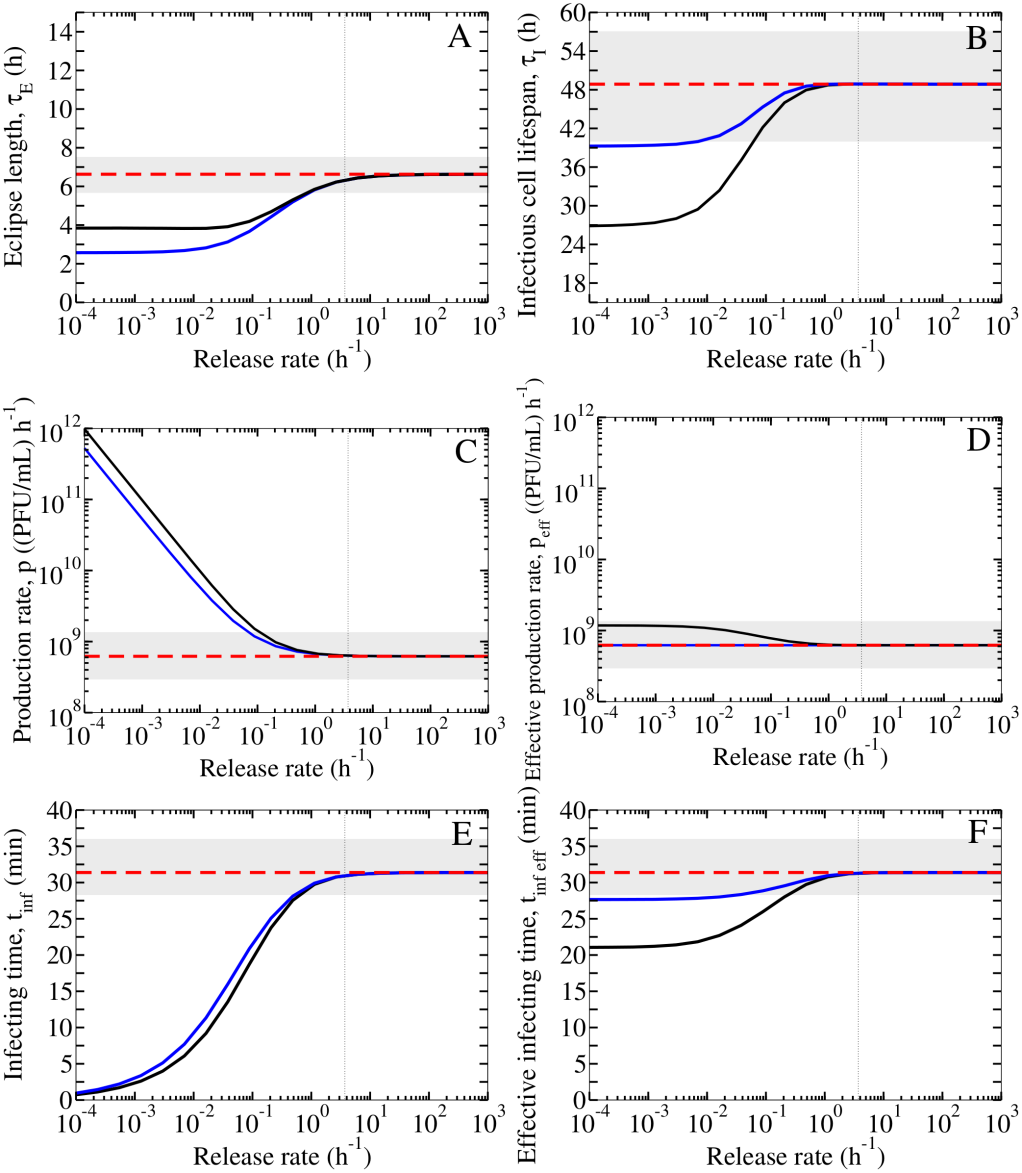
Comparing parameter estimates between the simple MM and release MM. The simple MM base parameters of an in vitro infection with the 2009 pandemic influenza A (H1N1) virus strain [20] are shown (red) with 95% confidence intervals (grey band). For the release MM, the parameter estimates obtained (*y*-axis) as a function of the viral release rate (*x*-axis) are shown for the case when all parameters (*p*, *β*, *c*, *τ*_*E*_, *τ*_*I*_) are estimated (black), or for the case with the constraint *p*_release_ = *p*_simple_(*r* + *c*)/*r* (blue). The critical free virus release rate, *r*_*f*_ = 3.7 h^−1^, is indicated (vertical dotted line).

Fig 4 shows the infection parameters predicted by the release MM as a function of the release rate, *r*. If free virus release is rapid and effective (*r* > *r*_*f*_), the simple MM returns the same parameter estimates as predicted by the release MM, and use of the simple MM is appropriate in this circumstance. If free virus release is slow and inefficient (*r* < *r*_*f*_), the simple MM no longer correctly estimates the infection parameters.

In particular, Fig 4 shows that the simple MM significantly overestimates the eclipse length, i.e., the time a cell remains infected but not yet producing free virus (Fig 4A), and infectious cell lifespan, i.e., the time a cell remains infectious and producing free virus (Fig 4B), and underestimates the virus production rate (Fig 4C). The remaining fitted parameters do not significantly differ between the MMs (S4 Fig).

As previously described, the interpretation of the eclipse length differs between the two MMs and this is reflected in the estimate of *τ*_*E*_ obtained from the fits. In Fig 3A, the SC assay shows an approximately 6.7 h delay before the growth of free virus is measured. If virus release is assumed to be rapid (*r* > *r*_*f*_), the release MM ascribes the full delay to the eclipse length which represents the viral replication time. On the other hand, if virus release is assumed to be slow (*r* < *r*_*f*_), the release MM ascribes only part of the delay to the eclipse length (time for viral replication), while the rest is ascribed to the time required to release virus. Consequently, as the release rate decreases, less and less of the delay is ascribed to virus replication (the estimated eclipse length decreases) while the time required to release virions increases more and more. This highlights the release MM’s ability to ascribe the delay discriminately to each process, i.e., separately to virus replication and release. In the simple MM, the full delay is ascribed to the eclipse length, since parameter *τ*_*E*_ accounts for both the viral replication time and release time. Though it appears that the simple MM overestimates the eclipse length (Fig 4A, red vs black), this is simply a difference in interpretation of *τ*_*E*_ between these MMs.

As the virus release rate is decreased in the release MM, the production rate (*p*) deviates from the simple MM’s estimate in Fig 4C. Once again, parameter *p* requires a different interpretation in each MM because it represents different processes. In the simple MM, *p* represents the rate at which free infectious virus is produced and (instantaneously) released into the medium by an infectious cell, but in the release MM it represents the rate at which cell-associated, bound virions are produced onto the cell’s surface. If the release of these cell bound virions is slow, some will lose infectivity (at rate *c*) before they can be released into the medium, resulting in an effective rate of free virus production into the medium which could be much less than the rate at which cell-associated bound virus are produced onto the cell’s surface. It is easy to show (see Methods) that only a fraction *r*/(*r* + *c*) of all virions produced onto the cell’s surface will ultimately be released as free virions. This term also naturally appears in the equations for the basic reproductive number (Eq (8)) and the effective infecting time (Eq (9)). Therefore, it is the effective rate of free virus production, *p*_eff_ = *p* ⋅ *r*/(*r* + *c*), rather than the rate of bound virus production, *p*, in the release MM which should be compared to the free virus production rate *p* which appears in the simple MM.

**Fig 5:**
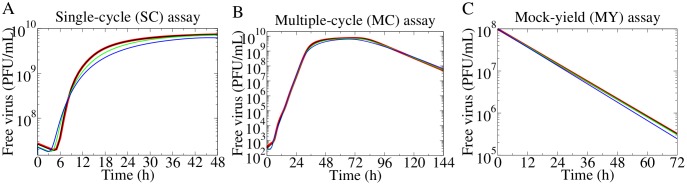
Simultaneous fits of the release MM to the simulated SC, MC, MY titers with a constraint on virus production. As in Fig 3, but with a constraint on the virus production rate, i.e., *p*_release_ = *p*_simple_(*r* + *c*)/*r* where *p*_simple_ = 6.28 × 10^8^(PFU/mL) ⋅ h^−1^.

With this in mind, we repeated the fit of the release MM to the data simulated with the simple MM while fixing the effective production rate in the release MM equal to the virus production rate in the simple MM, i.e., *p*_release_ = *p*_simple_(*r* + *c*)/*r* = (6.28 × 10^8^(PFU/mL) ⋅ h^−1^)(*r* + *c*)/*r*. From the fitted curves in Fig 5A, the SC titer recovers the familiar plateau and no longer exhibits a bump as was the case in Fig 3A when no constraint was applied to *p*. As the release rate decreases, the fits cannot capture the steep upslope of the SC titer. The fits performed without a constrained production rate yields the lowest Akaike information criterion (AIC_C_, see Methods), but does not reproduce the plateau in the SC assay. The AIC_C_ is an index that ranks MMs, where the minimum score indicates the MM that gives a better fit to the data, while guarding against overfitting by imposing a penalty against MMs that have a higher number of fitted parameters. For the fits performed with a constrained production rate, a higher AIC_C_ is obtained despite the one parameter reduction, but the plateau in the SC assay is recapitulated.

Fig 4 shows the parameter values predicted by the release MM under this constraint. When virus release is rapid (*r* > *r*_*f*_), the simple MM estimates the same parameters as predicted by the release MM. When virus release is slow (*r* < *r*_*f*_), the simple MM estimates the same effective production rate as predicted by the release MM in either analysis. For the remaining parameters (*τ*_*E*_, *τ*_*I*_, *t*_inf eff_), the simple MM either: (1) overestimates them compared to the predictions by the release MM if there is no constraint on *p*; or, (2) estimates the same values as predicted by the release MM with *p* constrained, except for the eclipse length due to the differing definitions between the two MMs.

### Characterizing NAI efficacy in the context of explicit vs implicit release

We used the simple and release MMs to simulate infections in the absence (untreated) and presence (treated) of NAIs, and compared the two MM’s predictions for the effect of NAIs on free virus titer. In both MMs, the NAI efficacy is expressed as a fractional inhibition, *ε*, which ranges between 0 (no inhibition) and 1 (total inhibition). In the simple MM, virus release is modelled implicitly as a part of the virus production rate *p*, so the action of NAIs is represented as inhibiting the rate of virus production, i.e., (1 − *ε*_*p*_)*p* [8–11]. In the release MM, the NAIs can be correctly implemented as inhibiting the release rate directly, i.e., (1 − *ε*_*r*_)*r*. The subscript on the symbol for efficacy denotes the infection parameter upon which the drug acts.

**Fig 6:**
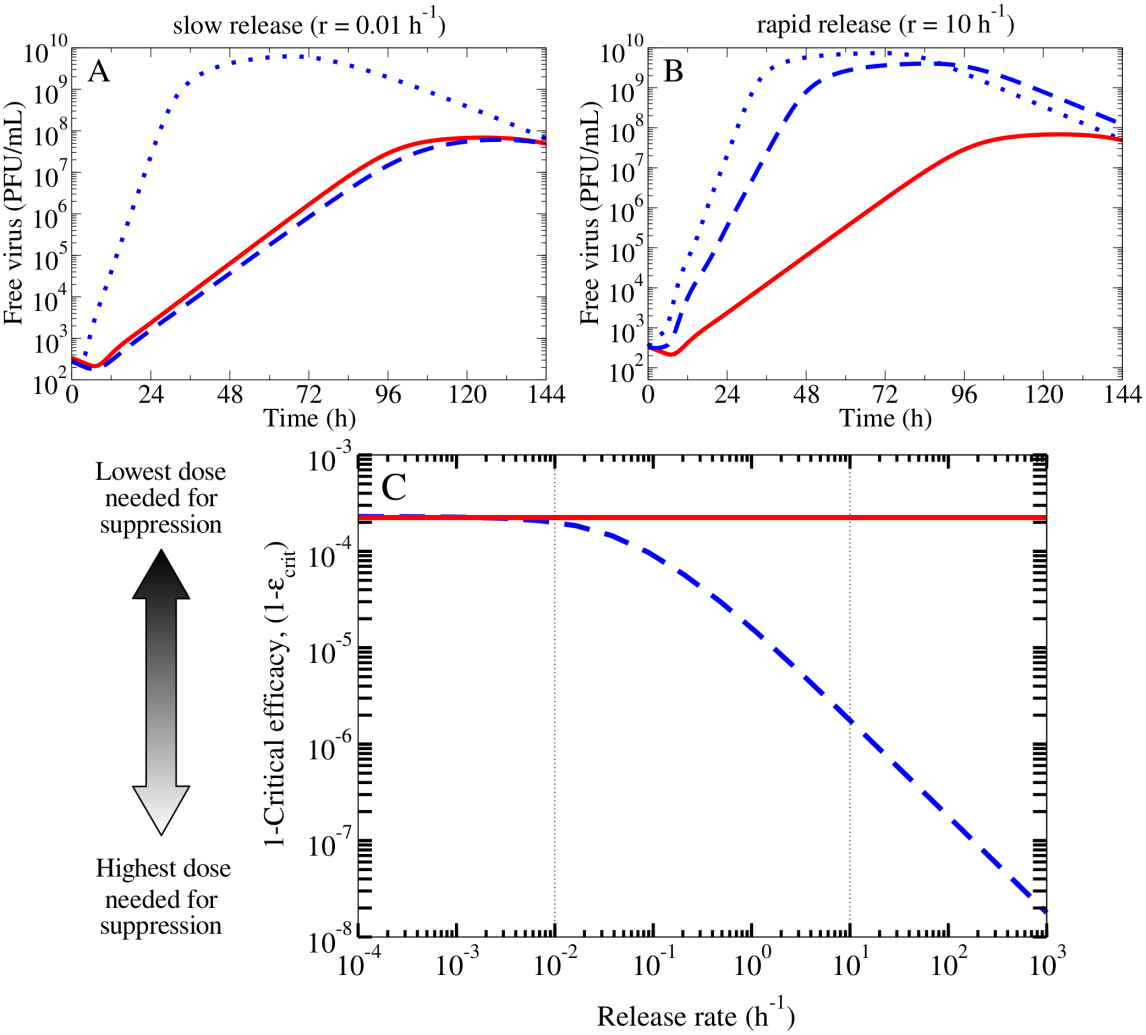
Comparing the effect of NAIs in the simple and release MMs. (A) For slow virus release (*r* = 0.01 h^−1^), both MMs predict a similar viral kinetic time course for treatment with NAI at a constant efficacy of 0.99, compared to the untreated infection (blue dotted line). (B) With faster virus release (*r* = 10 h^−1^), the simple MM predicts more significant viral yield suppression than the release MM for the same NAI efficacy. (C) The (1 − *ε*_crit_) of NAIs in the simple MM (red solid line) and the release MM (blue dashed line) is shown as a function of the release rate. The vertical dotted lines in (C) indicate the release rates used in (A) and (B).

We simulated MC infections under NAI therapy where drug was applied at the start of the infection, at a constant efficacy of 0.99 in both MMs. If the virus release is slow (*r* = 0.01 h^−1^), Fig 6A shows that NAI therapy in either MM reduces and delays the free virus peak titer by the same degree. If the virus release is fast (*r* = 10 h^−1^), the simple MM predicts that NAI therapy results in a greater reduction and delay in the free virus peak titer than that predicted by the release MM for an equal efficacy (Fig 6B). As a consequence, if a particular viral kinetics time course under NAI therapy is observed, and the efficacy is estimated, the simple MM would underestimate that efficacy. That is, if virus release is fast, the simple MM suggests that a lower efficacy of drug is required to achieve a given infection kinetics under NAI therapy, thereby also underestimating the dose required to achieve that effect.

For a comparison of the differential effect of NAIs in either MM over the entire range of release rates considered, we used a proxy to concisely represent the above information. We calculated the critical efficacy (*ε*_crit_) from the fitted parameters. The critical efficacy is defined as the minimum efficacy required to cause the suppression of infection, i.e., reduce *R*_0_ below its threshold value of 1. Fig 6C shows (1 − *ε*_crit_) from the simple MM (Eq (10)) and the release MM (Eq (11)) as a function of the explicit release rate. At high release rates, the simple MM predicts higher values of (1 − *ε*_crit_), indicating that a lower efficacy of NAIs is needed to achieve the total suppression of infection than predicted by the release MM. From this, we can see that the simple MM underestimates NAI efficacy if virus release is fast, where the error in estimation is largest as the virus release rate increases. Interestingly, the simple MM predicts NAI efficacies similar to those predicted by the release MM if release is slow. This is in contrast to the finding that the simple MM predicts the same estimates of the infection parameters as the release MM only if virus release is rapid.

### Exploring the difference between NAIs and polymerase inhibitors in the context of the release MM

**Fig 7:**
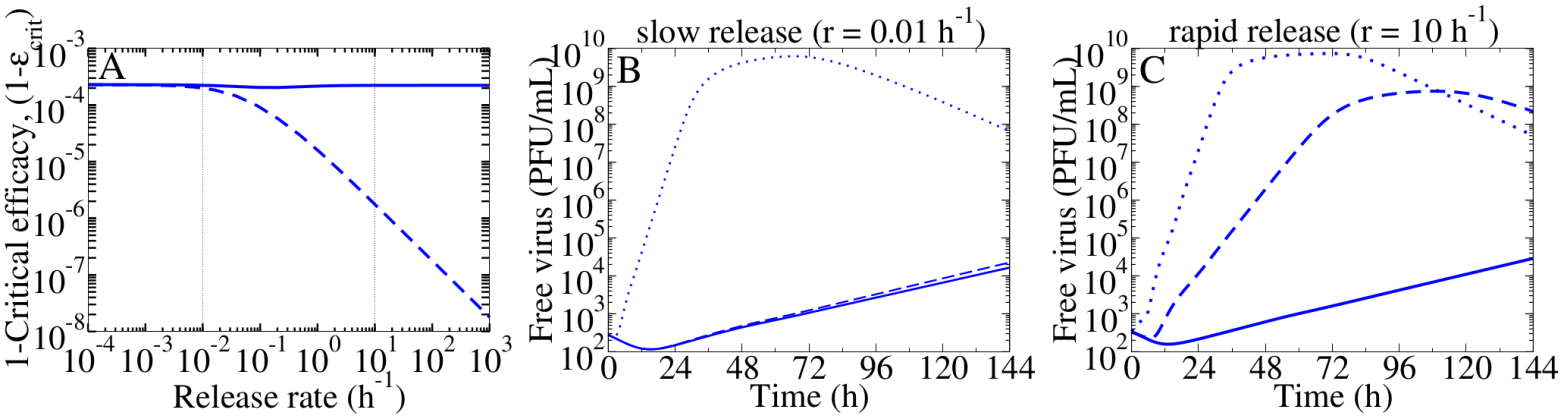
Comparing antivirals that inhibit release to antivirals that inhibit production in the release MM. (A) The (1 − *ε*_crit_) of an antiviral that inhibits virus production (*ε*_*p*_, solid) is compared to that of an antiviral that inhibits release (*ε*_*r*_, dashed). The vertical dotted lines indicate the release rates in (B) and (C). (B) With slow release (*r* = 0.01 h^−1^), the release MM predicts a similar viral kinetic time course for treatment with either antiviral at an efficacy of 0.999. The untreated infection is shown (dotted). (C) With rapid release (*r* = 10 h^−1^), the release MM predicts that an antiviral that inhibits virus production suppresses viral yield significantly more one inhibiting virus release, for the same efficacy.

The release MM provides us with the opportunity to compare antivirals that inhibit virus production (acting on *p*) to those that inhibit virus release (acting on *r*). Fig 7 shows that if the true influenza A virus release rate is less than 0.01 h^−1^, both classes of antivirals at the same efficacy will have an equal effect on the infection. On the other hand, if the virus release rate is greater than 0.01 h^−1^, the release MM predicts that an antiviral inhibiting virus production requires a lower dose (is more effective) to achieve suppression of infection than an antiviral that inhibits virus release. Regardless of the true influenza A virus release rate, an antiviral acting to suppress viral replication/production is predicted to always suppress free virus titer to an extent equivalent to or greater than an antiviral inhibiting virus release.

## Discussion

Mathematical models (MMs) describing the kinetics of influenza A virus infections in vitro and in vivo typically represent the release of cell-bound progeny virions from the surface of productively infected cell implicitly. Specifically, the rate of virus release gets incorporated into aggregate, generic parameters which stand in for the combined kinetics of virus replication, assembly, budding, and release. Importantly, when these simple MMs are used to characterize treatment of influenza A virus infections with NAIs [8–11], they represent the action of NAIs as suppressing virus production, or rather suppressing the combined kinetics of replication, assembly, budding and release embodied in the simple MM’s parameter designated as the rate of free virus production.

Herein we developed a variant of the simple MM, hereafter the release MM, that includes an explicit description of virus release. The release MM accounts for two distinct populations of infectious virus, bound and free virions, with cell-associated, *bound* virions being released into the medium as *free* virions at release rate *r*. Unfortunately, the rate at which influenza A virus progeny is released from the surface of the cell that produces them is not well-known. Using the release MM, we explored a wide range of virus release rates to assess the impact of this additional, unknown MM parameter on infection parameter estimates, and in particular the estimation of NAI efficacy, compared to those obtained with the simple MM.

Mathematically, in the absence of antiviral therapy, the release MM reduces to the simple MM if the virus release rate is sufficiently large. Our work shows that for realistic parameter values in the context of an influenza A virus infection in vitro, this occurs when the virus release rate is greater than ∼4 h^−1^. If virus release is more efficient than this, release does not significantly affect infection kinetics or free virus yield, making the simple MM a valid approximation of the release MM. If virus release is less efficient than this, it will affect infection kinetics and free virus yield. In fact, if virus release is slower than ∼0.1 h^−1^, the two MMs statistically significantly disagree in their estimates of some infection parameters, specifically the virus production rate, the eclipse length, and the lifespan of infectious cells (i.e., the duration of virus production). This is because although the simple and release MMs share most of the same structure, the implementation of implicit versus explicit virus release imparts onto each MM a different interpretation of the infection parameters they have in common.

Biologically, if the release of virions into the medium is slow such that the release rather than the production of virus is a kinetic bottleneck, a portion of the bound virions produced onto the cell’s surface will lose infectivity before they can be released as free virions into the medium. For the release MM, we show that only a fraction *r*/(*r* + *c*) of the bound virions produced onto the cell’s surface are ultimately released into the medium as free virions, wherein *r* is the virus release rate and *c* is the rate at which virions lose infectivity. If *r* is large relative to *c*, *r*/(*r* + *c*) ≈ 1, and all produced virions are released as free virions. In fact, we found that the free virus production rate (*p*) estimated by the simple MM agrees with the effective free virus production rate (*p* ⋅ *r*/(*r* + *c*)) estimated by the release MM. Furthermore, when we repeated parameter estimation while specifically imposing the constraint that *p*_simple_ = *p*_release_ *r*/(*r* + *c*), the two MMs agreed in their estimation of all parameters except for the length of the eclipse phase. While the eclipse phase length in the simple MM measures the delay from the infection of a cell until free virus release, in the release MM it measures the time elapsed between infection of the cell and the appearance of progeny virions bound to the infected cell’s surface, specifically excluding the time required for their release as free virus. For a given experimentally observed delay in the appearance of free virions, the simple MM can only ascribe it to the eclipse length whereas the release MM must distribute its duration between the eclipse length and release rate. This should be kept in mind when interpreting estimates of the eclipse phase length obtained using the simple MM.

When it comes to representing the mode of action of NAIs, the simple MM is restricted to inhibiting the aggregate free virus production rate, whereas the explicit MM can specifically inhibit the rate of virus release. If virus release is slow (*r* < *c*) such that release rather than production limits free virus yield, the two MMs predict the same infection kinetics for the same NAI efficacy. In the release MM, if virus release is rapid such that virus production rather than release is the bottleneck, imposing the limit on free virus yield (*r* ≫ *c*), even a large reduction in the release rate has a modest impact on virus yield until it is reduced to the point where *r* ≈ *c*. For this reason, if virus release is rapid, NAIs in the release MM have a weaker suppressive effect than in the simple MM for the same efficacy. In other words, for a given experimentally observed reduction in free viral yield, the simple MM will underestimate the true NAI efficacy required to effect this reduction. This error increases with increasing virus release rates. Underestimating antiviral efficacy in turn, leads to underestimating the NAI dosage required to achieve a specific therapeutic outcome, e.g., suppression of infection. Underdosing prolongs the overall duration of infection and the number of genomes produced, which could increase the likelihood of developing resistance to NAIs. In Japan, NAI-resistant A/H3N2 strains were detected from children who received oseltamivir, and underdosing had been thought to be a contributing factor [23, 24].

Our results demonstrate the need to estimate the rate of influenza A virus release in order to correctly interpret results obtained from the simple MM. However, directly measuring the virus release rate is difficult for a number of reasons. An uninfected cell will express a number of sialic acid receptors on its surface. After successful infection by an influenza A virus, virus replication gets underway and ∼1 h–3 h post-infection, the cell begins to express increasing levels of viral NA on its surface [25, 26]. These surface viral NAs proceed to cleave more and more of the cell’s sialic acid receptors as time elapses. As such, the sialic acid receptor landscape seen by progeny virions budding onto the cell’s surface is much less dense than than that seen by infecting influenza A virions entering an uninfected cell. For this reason, detachment rates of influenza A virus measured in studies using uninfected cells (where endocytosis and infection are blocked) cannot be thought of as estimates of the virus release rates, but could possibly be providing a lower bound for that estimate. For example, Nunes-Correia et al. [27] found detachment rates of 0.36 h^−1^ –11 h^−1^ for influenza A PR8 (H1N1) virus interacting with uninfected MDCK cells. Since there are more receptors on uninfected cells, we expect that the true virus release rate would be greater than that found by Nunes-Correia et al. However, the study by Nunes-Correia et al. was performed on MDCK cells which express far fewer *α*-2,6 cell receptors than that found on the surface of epithelial cells lining the human respiratory tract (RT) [21]. Due to this lower level of *α*-2,6 on MDCK cells, these rates likely overestimate the biologically relevant detachment rates and therefore cannot be thought of as a true lower bound. In [28], other key studies of influenza A virus attachment and detachment from uninfected cells were highlighted, with detachment rates estimates as low as 0.029 h^−1^ and as high as 11 h^−1^. However, these were estimated with different influenza A viruses interacting in non-cell systems (e.g., influenza A PR8 (H1N1) virus interacting with bovine brain lipid membrane, or influenza A (H3N2) and duck influenza A (H5N3) virus strains binding to gangliosides) which might not reflect the cell receptor composition of an infected human RT epithelial cell.

Our release MM predicts that at equal efficacy, treatment with an antiviral against virus production suppresses free virus yield to a greater extent than an antiviral against release if the release rate is greater than 0.01 h^−1^. This is consistent with the observation that favipiravir, a new antiviral which inhibits viral polymerase, is more potent and effective than NAIs [29] (refer to [30] for a review). Therefore, it is possible that influenza A virus strains that are more strongly suppressed by favipiravir than NAIs have a release rate greater than ∼ 0.01 h^−1^. In our view, the best way to obtain an estimated lower bound for the release rates, would be to repeat the attachment/detachment study performed by Nunes-Correia et al. using MDCK*α*2,6 cells which express *α*-2,6 sialic acid cell receptors at levels similar to those found on human RT epithelial cells.

In our work, we assume that the virus release rate is constant throughout the entirety of the cell’s infectious phase. As discussed above, the amount of cell surface NA increases and the number of cell receptors decreases as the infection progresses, such that the rate of virus release should in fact depend on the time elapsed since the cell became infected. One possible MM extension is the integration of an age of infection-dependent release rate, where a simple step function could switch from a low to high release rate as the infection progresses. Without appropriate quantitative data, however, there is little to justify the added complexity to the MM.

In conclusion, we have highlighted the importance of quantifying the influenza A virus release rate and the implications of an implicit representation of virus release in the simple MM. In future analyses of virus replication kinetics, it would be desirable to design an additional in vitro assay, to expand the current suite of assays (i.e., MC, SC, MY), which could provide an accurate, independent estimate of the virus release rate. It remains to be determined whether or not viral release acts as a kinetic bottleneck in influenza A virus replication.

## Methods

### Mathematical models of influenza A viral infection kinetics

Influenza A virus infections in vitro were numerically simulated with a multi-compartment ordinary differential equation (ODE) model as previously described and validated in [20–22]. Throughout, we refer to it as the simple MM in order to distinguish it from a variant we call the release MM. The release MM shares almost all equations with the simple MM, but possesses one additional parameter, the virus release rate *r*, and one additional equation to distinguish cell-associated bound virus from released free virus. The equations common to both MMs describes the flow of cells through the various phases of infection as follows

$$\begin{array}{cc} \frac{dT}{dt} & =-\beta TV \end{array}$$ (1)

$$\begin{array}{cc} \frac{dE_{1}}{dt} & =\beta TV-\frac{n_{E}}{\tau_{E}}E_{1} \end{array}$$ (2)

$$\begin{array}{cc} \frac{dE_{i}}{dt} & =\frac{n_{E}}{\tau_{E}}E_{i-1}-\frac{n_{E}}{\tau_{E}}E_{i}\;\;\text{for}\;i=\left(2,\cdots ,n_{E}\right) \end{array}$$ (3)

$$\begin{array}{cc} \frac{dI_{1}}{dt} & =\frac{n_{E}}{\tau_{E}}E_{n_{E}}-\frac{n_{I}}{\tau_{I}}I_{1} \end{array}$$ (4)

$$\begin{array}{cc} \frac{dI_{j}}{dt} & =\frac{n_{I}}{\tau_{I}}I_{j-1}-\frac{n_{I}}{\tau_{I}}I_{j}\;\;\text{for}\;j=\left(2,\cdots ,n_{I}\right) \end{array}$$ (5)

where fractional (∈[0, 1]) populations of uninfected target cells, *T*, are infected at infection rate *β* times the concentration of free, infectious virus, *V* (in PFU/mL). Newly infected cells enter the eclipse phase, $E_{i=1,...,n_{E}}$, and transition into the infectious phase, $I_{j=1,...,n_{I}}$, after an average time *τ*_*E*_ and *τ*_*I*_ have elapsed, respectively. The eclipse (or infectious) phase is divided into *n*_*E*_ (or *n*_*I*_) compartments such that the time spent by cells in the phase follows an Erlang distribution [20, 31, 32].

The two MMs differ only in their equations for infectious virus, wherein the release MM includes one additional variable and one new parameter to explicitly account for the release of cell-bound virus as free virus

$$\begin{array}{ll} \text{Simple MM} & \text{Release MM}\\ & \frac{\text{d}V_{\text{b}}}{\text{d}t}\;=p\sum_{i=1}^{n_{I}}I_{i}-cV_{\text{b}}-rV_{\text{b}} \end{array}$$ (6)

$$\begin{array}{ll} \frac{\text{d}V}{\text{d}t}=p\overset{n_{I}}{\underset{i=1}{\sum }}I_{i}-cV\; & \frac{\text{d}V}{\text{d}t} \end{array}\;=rV_{\text{b}}-cV\;.$$ (7)

In the simple MM (without explicit viral release), free infectious virus *V* is produced directly into the medium by infectious cells (*I*) at a rate *p*, and lose infectivity at rate *c*. In the release MM, cell-bound virus *V*_b_ is produced onto the surface of infectious cells at a rate *p*, and also lose infectivity at rate *c* while bound to the cell. The infectious bound virus is released from the cell surface at rate *r* and enters the medium as free virus, which also lose infectivity at rate *c*. The release MM distinguishes virus release from any other processes that are encompassed by the virus production rate. Our base parameter values for the simple MM are listed in Table 1, and were taken from [20] where the kinetics of an in vitro infection with the 2009 pandemic influenza A/Québec/144147/09 (H1N1) virus strain was analyzed.

Two important secondary parameters, the basic reproductive number (*R*_0_) and the infecting time (*t*_inf_), can be derived from the MM parameters. The infecting time represents the time it takes for one infectious, virus-producing cell to infect one other cell in a fully susceptible population of cells. It has been described in [31–33] and is given by

$$\begin{array}{c} t_{\text{inf}}=\sqrt{\frac{2}{p\beta }}\;. \end{array}$$

The infecting time is reported in lieu of parameters that are measured in plaque-forming units, such as *p* and *β*, because of the undetermined, relative relationship between infectious virus and experimental measures of virus infectivity such as 50% infectious dose (TCID_50_, CCID_50_) or plaque-forming unit (PFU) [7, 34].

The basic reproductive number is another commonly reported quantity and it represents the number of secondary infections caused by one infectious cell in a population of fully susceptible cells. If *R*_0_ is less than the threshold value of 1, the spread of infection is suppressed. The basic reproductive number for each MM is given by

$$\begin{array}{ll} \text{Simple MM} & \text{Release MM}\\ R_{0}=\frac{p\beta \tau_{I}}{c}\; & R_{0}\;=\frac{p\beta \tau_{I}}{c}\;\left[\frac{r}{r+c}\right]\;. \end{array}$$ (8)

### The effective production rate in the release MM

Assuming that the fraction of productively infectious cells is unchanged over a certain timescale of interest ($I=\bar{I}$), Eq (6) for bound virus in the release MM becomes

$$\begin{array}{c} \frac{dV_{b}}{dt}=p\bar{I}-\left(r+c\right)V_{b}\;. \end{array}$$

Further assuming that no bound virus is initially present, integrating the above equation yields:

$$\begin{array}{c} V_{b}\left(t\right)=\frac{p\bar{I}}{r+c}[1-e^{-(r+c)t}]. \end{array}$$

Substitution of *V*_b_(*t*) into the differential equation describing free virus yields:

$$\begin{array}{c} \frac{dV}{dt}=\underset{p_{\text{eff}}}{\underset{︸}{\frac{r}{r+c}p}}\bar{I}[1-e^{-(r+c)t}]-cV \end{array}$$

where the correction factor $\frac{r}{r+c}$ on the virus production rate accounts for bound virus which loses infectivity before it can be released. Thus, the effective production rate in the release MM is given by $p_{\text{eff}}=p\frac{r}{r+c}$.

We use the effective production rate to correct the expression of the infecting time. This corrected parameter is called the effective infecting time,

$$\begin{array}{c} t_{\text{inf}}{\;}_{\text{eff}}=\sqrt{\frac{2}{p_{\text{eff}}\beta }}=\sqrt{\frac{2}{p\beta }\frac{\left(r+c\right)}{r}}\;. \end{array}$$ (9)

Such a correction is necessary since the infecting time (*t*_inf_) will deviate from the intended physical meaning for release rates that are less than 0.1 h^−1^. This is because the expression for *t*_inf_ was originally derived under the assumption that the rate of loss of virion (approximately 0.1 h^−1^) could be neglected.

### Modelling antiviral effect

When infections were treated with a neuraminidase inhibitor (NAI), the drug efficacy *ε* was applied to either the production rate in the simple MM, as (1 − *ε*_*p*_)*p*, or to the release rate in the release MM, as (1 − *ε*_*r*_)*r*. The efficacy (*ε*_*p*_ or *ε*_*r*_) takes on values between 0 (no efficacy) and 1 (maximum efficacy), where a more efficacious drug leads to greater reduction of either the virus production or release rate. Treatment was always applied at the start of infection (*t* = 0 h).

In each MM, treatment with NAIs is given by:

$$\begin{array}{ll} \text{Simple MM} & \text{Release MM}\\ & \frac{\text{d}V_{\text{b}}}{\text{d}t}\;=p\sum_{i=1}^{n_{I}}I_{i}-cV_{\text{b}}-\left(1-\varepsilon_{r}\right)rV_{\text{b}}\\ \frac{\text{d}V}{\text{d}t}\;=\left(1-\varepsilon_{p}\right)p\sum_{i=1}^{n_{I}}I_{i}-cV & \frac{\text{d}V}{\text{d}t}\;=\left(1-\varepsilon_{r}\right)rV_{\text{b}}-cV \end{array}$$

Additionally, the release MM allows for treatment with an antiviral which targets the virus production rate, separate from that which targets the virus release rate. In the release MM, treatment with an antiviral which blocks virus production is given by:

$$\begin{array}{l} \text{Release MM}\\ \frac{\text{d}V_{\text{b}}}{\text{d}t}\;=\left(1-\varepsilon_{p}\right)p\sum_{i=1}^{n_{I}}I_{i}-cV_{\text{b}}-rV_{\text{b}}\;. \end{array}$$

We define the critical efficacy (*ε*_crit_) as the minimum efficacy required to reduce the basic reproductive number below 1. Re-arranging the above when *R*_0_ = 1 yields the following expressions for critical efficacies in the simple MM

$$\begin{array}{c} \varepsilon_{p\text{crit}}=1-\frac{c}{p\beta \tau_{I}}, \end{array}$$ (10)

and in the release MM

$$\varepsilon_{p\text{crit}}=1-\frac{c}{p\beta \tau_{I}}\frac{\left(c+r\right)}{r}$$ (11)

$$\varepsilon_{r\text{crit}}=1-\frac{1}{\left[\frac{p\beta \tau_{I}}{c}-1\right]}\frac{c}{r}\;.$$ (12)

### Numerically solving transformed equations

**Fig 8:**
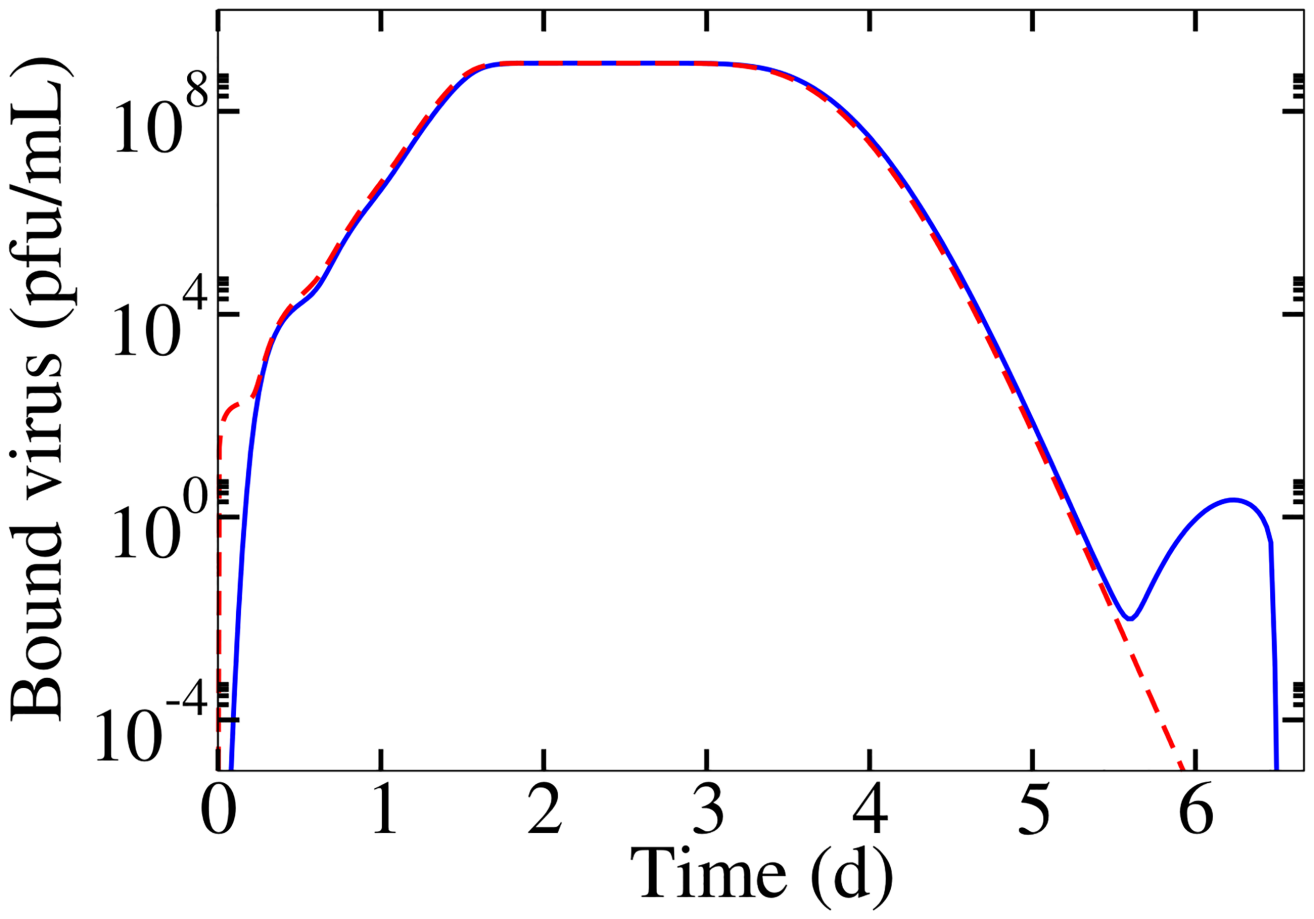
Instabilities in numerically solving model ordinary differential equations (ODE). Numerically solving ODEs in Eqs (1)–(7) (blue) results in instabilities, however solving transformed Eqs (13)–(19) (red) resolves the issue.

Numerically solving the MM in the form of Eqs (1)–(7) with the lsode solver in Octave 3.8.1 suffered from instabilities (Fig 8). To resolve this issue, a change of variables was done to transform Eqs (1)–(7). For example, Eq (1) was transformed by introducing variables $L_{V}=\ln(\frac{V}{V_{\text{res}}})$ and $L_{T}=\ln(\frac{T}{N})$ where *V*_res_ and *N* were rescaling factors required to maintain a dimensionless argument within the natural logarithm. For example,Eq (1) becomes

$$\begin{array}{c} \frac{dL_{T}}{dt}=\frac{dL_{T}}{dT}\frac{dT}{dt}=-\frac{e^{-L_{T}}}{N}\beta TV=-\frac{e^{-L_{T}}}{N}\beta \left(Ne^{L_{T}}\right)\left(V_{\text{res}}e^{L_{V}}\right)\;. \end{array}$$

The resulting full set of transformed equations is given by

$$\begin{array}{cc} \frac{dL_{T}}{dt} & =-\beta V_{\text{res}}e^{L_{V}} \end{array}$$ (13)

$$\begin{array}{cc} \frac{dL_{E_{1}}}{dt} & =\beta V_{\text{res}}e^{L_{T}+L_{V}-L_{E_{1}}}-\frac{n_{E}}{\tau_{E}} \end{array}$$ (14)

$$\begin{array}{cc} \frac{dL_{E_{i}}}{dt} & =\frac{n_{E}}{\tau_{E}}e^{L_{E_{i-1}}-L_{E_{i}}}-\frac{n_{E}}{\tau_{E}} \end{array}$$ (15)

$$\begin{array}{cc} \frac{dL_{I_{1}}}{dt} & =\frac{n_{E}}{\tau_{E}}e^{L_{E_{n_{E}}}-L_{I_{1}}}-\frac{n_{I}}{\tau_{I}} \end{array}$$ (16)

$$\begin{array}{cc} \frac{dL_{I_{i}}}{dt} & =\frac{n_{I}}{\tau_{I}}e^{L_{I_{i-1}}-L_{I_{i}}}-\frac{n_{I}}{\tau_{I}} \end{array}$$ (17)

$$\begin{array}{ll} \text{Simple MM} & \text{Release MM}\\ \frac{\text{d}L_{V}}{\text{d}t}\;=p\frac{N}{V_{\text{res}}}{\text{e}}^{-L_{V}}\sum_{i=1}^{n_{I}}{\text{e}}^{L_{I_{i}}}-c & \frac{\text{d}L_{V_{b}}}{\text{d}t}\;=p\frac{N}{V_{\text{res}}}{\text{e}}^{-L_{V_{b}}}\sum_{i=1}^{n_{I}}{\text{e}}^{L_{I_{i}}}-c-r \end{array}$$ (18)

$$\begin{array}{ll} & \frac{\text{d}L_{V}}{\text{d}t}=r{\text{e}}^{L_{V_{b}}-L_{V}}-c \end{array}$$ (19)

Fig 8 shows that a change of variables resolved the errors in numerically solving the system of equations.

### Simulation of infections

#### Multiple-cycle, single-cycle and mock-yield simulated data

The multiple-cycle (MC), single-cycle (SC), and mock-yield (MY) in vitro assays in Figs 2, 3 and 5 (red) were simulated with the simple MM, using the base parameters in Table 1. The MC and SC assays were initiated with a population of fully susceptible cells (*T* = 1) that were infected with concentrations of the initial inoculum according to $V_{0}=\frac{c}{\beta }\left(\text{MOI}\right)$, where the MOIs were 5 × 10^−5^ PFU/cell and 4 PFU/cell, respectively. The MY assay was initiated with an initial virus inoculum of *V*_0_ = 10^8^ PFU/cell in the absence of cells (*T* = 0). The free virus titer from this suite of in vitro assays served as our simulated data with which the release MM was fitted.

In Fig 2 (black), infections were simulated with the release MM where the initial conditions and parameters were identical to the MC simulation in the simple MM, except that the release rate was varied as indicated.

#### Fitting the release MM to the simple MM-simulated data

Simultaneous fits of the release MM to the SC, MC, and MY viral yield simulated datasets were performed with the Nelder-Mead minimization method (nelder_mead_min from version 1.5.2 of the optim package in Octave 4.0.3). The fits minimized the combined sum-of-squared residuals per point (SSR/pt):

$$\begin{array}{c} \text{combined}\;\text{SSR}/\text{pt}=\text{SSR}/{\text{pt}}_{\text{SC}}N_{\text{SC}}+\text{SSR}/{\text{pt}}_{\text{MC}}N_{\text{MC}}+\text{SSR}/{\text{pt}}_{\text{MY}}N_{\text{MY}} \end{array}$$

where $\text{SSR}/\text{pt}=\frac{1}{N}\overset{N}{\underset{i=1}{\sum }}{\text{log}}_{10}{\left(\frac{V_{i}^{\text{data}}}{V_{i}^{\text{expMM}}}\right)}^{2}$ is computed for each assay, and *i* is the number of points of simulated free virus from the simple MM ($V_{i}^{\text{data}}$) and release MM ($V_{i}^{\text{expMM}}$). Each term is weighted by the number of experimental measurements typically made when collecting free virus titer in each assay (*N*_SC_ = 17, *N*_MC_ = 12, *N*_MY_ = 4) [22].

In each fit, the release rate was fixed in the release MM, while the parameters *p*, *β*, *c*, *τ*_*E*_, *τ*_*I*_ were fitted. The remaining parameters *n*_*E*_, *n*_*I*_ were kept at their base values (Table 1). The initial conditions for each assay were as described in Section. In total, multiple fits were performed for release rates in the range 10^−4^ h^−1^ –10^3^ h^−1^. The resultant sets of fitted parameters are shown in Fig 4 (black) and S4 Fig.

In another analysis, a constraint was imposed on the release MM’s virus production rate $p_{\text{release}}=p_{\text{simple}}\frac{r+c}{r}$, where *p*_simple_ = 6.21 × 10^8^ (PFU/mL) ⋅ h^−1^, enforcing the effective production rate in the release MM to be equal to the production rate in the simple MM. In this case, only four parameters were fitted (i.e., *β*, *c*, *τ*_*E*_, *τ*_*I*_) for each value of the release rate that was explored. The resultant sets of fitted parameters are shown in Fig 4 (blue) and S4 Fig.

**Fig 9:**
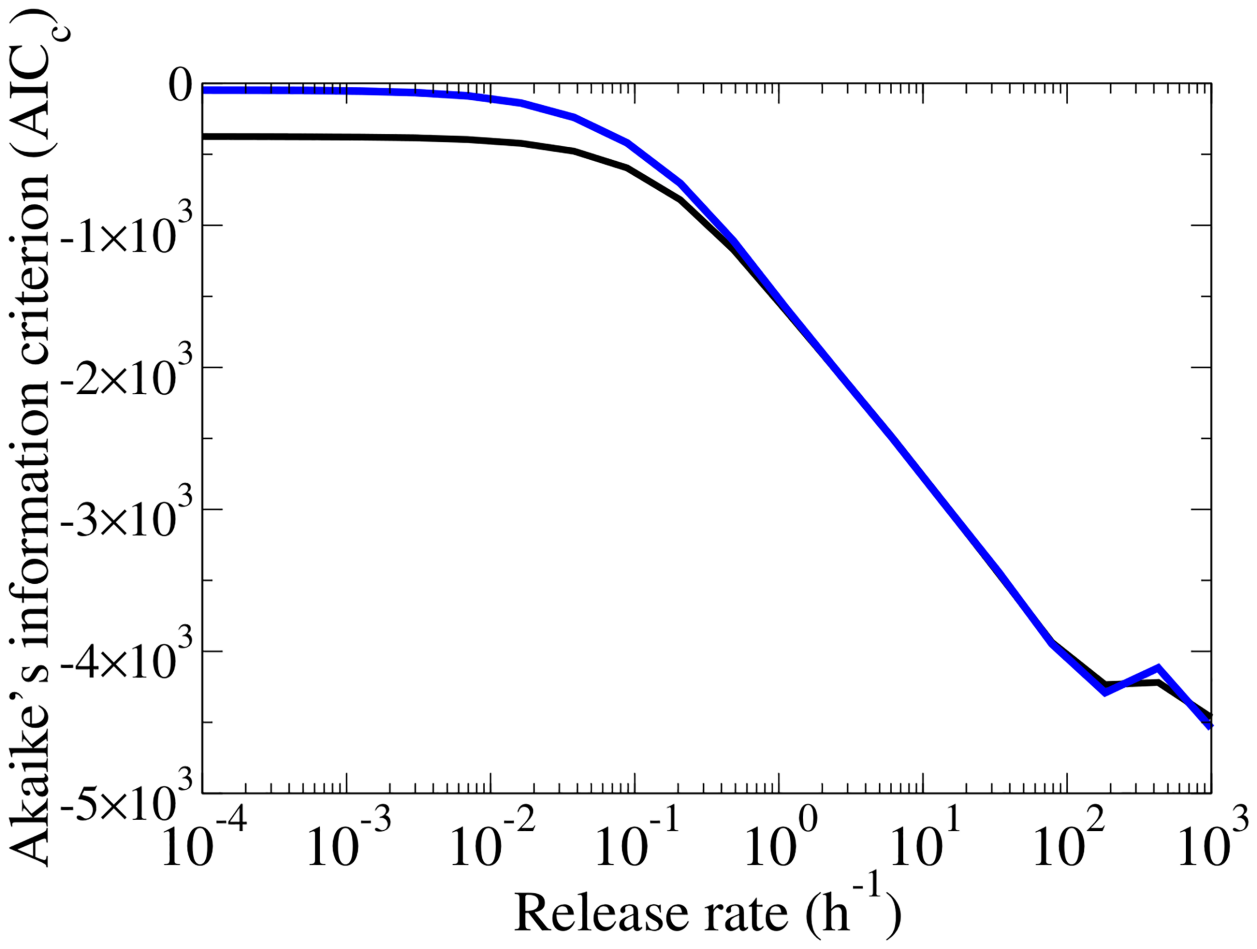
Comparing the AIC_C_ of release MM fits. The corrected Akaike information criterion (AIC_C_) is shown for fits to the release MM with a constraint on the virus production rate (*N*_par_ = 4, blue), and without a constraint (*N*_par_ = 5, black).

In Fig 9, we compared the analyses by computing the Akaike’s information criterion (corrected for small sample sizes) according to the expression given by [33], and based on [34]:

$$\begin{array}{c} AIC_{C}=N_{\text{pts}}\ln(\frac{SSR}{N})+\frac{2\left(N_{\text{par}}+1\right)N_{\text{pts}}}{N_{\text{pts}}-N_{\text{par}}-2} \end{array}$$

where $\frac{SSR}{N}$ is the combined SSR/pt from the fit, *N*_pts_ = 307 is the number of simulated data points and *N*_par_ is the number of parameters fitted. In the analysis without a constraint on the virus production rate *N*_par_ = 5, and in the analysis with the constraint *N*_par_ = 4.

### Treatment with antivirals

To illustrate that NAIs applied at identical efficacies in both MMs had a different effect on the free virus titer, we showed a MC infection in the absence of NAIs (untreated) and in the presence of NAIs (treated), as simulated by either MM in Fig 6A and 6B. The untreated and treated free virus titer were simulated in the simple MM using the base parameters (Table 1), where the treated infection received NAIs at the start of infection that were applied at an efficacy of *ε*_*p*_ = 0.99. With the constraint on virus production (Section), the release MM was fitted to the simple MM’s untreated free virus titer, and recapitulated these kinetics with a different set of parameters for each chosen release rate, *r* = 0.01 h^−1^, 10 h^−1^. Using these sets of parameters, the treated infections in the release MM were simulated with NAIs applied at an efficacy of *ε*_*r*_ = 0.99 at the start of infection.

In Fig 6C (blue), the critical efficacy of NAIs as a function of the release rate was computed (Eq (12)) from the sets of fitted parameters that were obtained from the release MM when a constraint was applied to the virus production rate. For comparison, the critical efficacy of NAIs from the simple MM (Eq (10)) was computed from the base parameters (Table 1), and was shown as a single value (straight line), independent of the release rate in Fig 6C (red).

In Fig 7A, the critical efficacy of antivirals against release (dashed) or production (solid) were computed with Eqs (12) and (11), respectively, using the same sets of fitted parameters that were determined with the release MM. To illustrate that the impact of antivirals which inhibit viral release differed from antivirals which inhibit virus production, untreated and treated MC infections were simulated in the release MM. For example, Fig 7B shows an untreated infection (blue dotted) that was simulated with the set of fitted parameters that was determined when the release rate was fixed to 0.01 h^−1^. The corresponding treated infections were simulated by applying an antiviral against production (solid) or release (dashed) at an efficacy of 0.999 at the start of infection. Fig 7C is the same, but a higher release rate (10 h^−1^) was used to simulate the infections.

## Supporting information

S1 Supporting Information Determination of critical release rates and parameters predicted by release MM. (PDF)

S1 Fig Determination of critical free and bound virus release rates, *r*_*f*_ and *r*_*b*_. (Left) The sum-of-squared residuals per point (SSR/pt) is computed between each free virus curve in the release MM, as the release rate is varied, and the simulated MC data in the simple MM (black). The critical free virus release rate, *r*_*f*_ = 3.72 h^−1^ (teal circle), corresponds to the variance of a mock-yield infection (dotted line). We also show the SSR/pt curve for various rates of loss of infectious virion that were explored (various colours). (Right) The peak value of bound virus titer as a function of the release rate is shown (black), where the maximum determines the critical bound virus release rate, *r*_*b*_ = 3 × 10^−3^ h^−1^ (purple circle). The various coloured lines correspond to various rates of loss of virion infectivity to show that *r*_*b*_ (dotted purple) strongly depends on other infection parameters.
(TIF)

S2 Fig The critical free virus release rate, *r*_*f*_, weakly depends on infection parameters. The critical free virus release rate, *r*_*f*_, weakly depends on the rate of loss of virion infectivity, production rate, infection rate, eclipse phase and infectious lifespan. The *r*_*f*_ when all parameters are at their base values is indicated with a horizontal dotted line.
(TIF)

S3 Fig The critical bound virus release rate, *r*_*b*_, depends on infection parameters. The critical bound virus release rate, *r*_*b*_, depends on the rate of loss of virion infectivity, production rate, infection rate, eclipse phase, and infectious lifespan. The *r*_*b*_ when all parameters are at their base values is indicated with a black circle.
(TIF)

S4 Fig The simple MM does not significantly misestimate the infection rate, rate of loss of virion infectivity, or the basic reproductive number. As in Fig 4, but showing the infection rate, rate of loss of virion infectivity, and the basic reproductive number.
(TIF)
