# Supplementary material for: Exploring virus release as a bottleneck for the spread of influenza A virus infection in vitro and the implications for antiviral therapy with neuraminidase inhibitors
Source: PLoS One. 2017 Aug 24;12(8):e0183621. doi: 10.1371/journal.pone.0183621 (PMC5570347; doi:10.1371/journal.pone.0183621)
Supplement: S1 Supporting Information — (PDF) [file pone.0183621.s001.pdf]

Supporting Information:
Exploring virus release as a bottleneck for the spread of influenza A virus infection in vitro and the implications for antiviral therapy with neuraminidase inhibitors

Laura E. Liao[1], Szymon Kowal[1], Daniel A. Cardenas[1], Catherine A. A. Beauchemin[1,2,*]

1 Department of Physics, Ryerson University, Toronto, Canada
2 Interdisciplinary Theoretical and Mathematical Sciences (iTHES, iTHEMs) research group at RIKEN, Wako, Japan
***** Corresponding author. Email: cbeau@ryerson.ca
PLOS ONE

July 24, 2017

Determination of critical release rates

The critical free virus release rate (r_f) is the minimum rate of viral release that causes the free virus titer in the release MM to overlap with the baseline free virus titer (i.e., the MC infection simulated by the simple MM) to a degree which they can be considered indistinguishable from each other. To quantitatively determine r_f, the distance between the free virus curves was computed using the sum-of-squared residuals per point (SSR/pt), where a smaller SSR/pt indicates a higher degree of similarity between curves. In Fig S1 (left), the SSR/pt as a function of release rate was computed between the baseline free virus and each free virus curve from the release MM (Fig 2A, red vs. black). The value of the SSR/pt which denoted overlapping curves was set to the variance associated with a mock-yield experiment, $\sigma^2_{\text{MY}} = (0.072 \text{ PFU/ml})^2 = 5.184 \times 10^{-3} \text{ (PFU/ml)}^2$ (horizontal dotted line; computed from [1], data not shown). When all infection parameters were at their base value, the critical free virus release rate was $r_f = 3.72 \text{ h}^{-1}$.

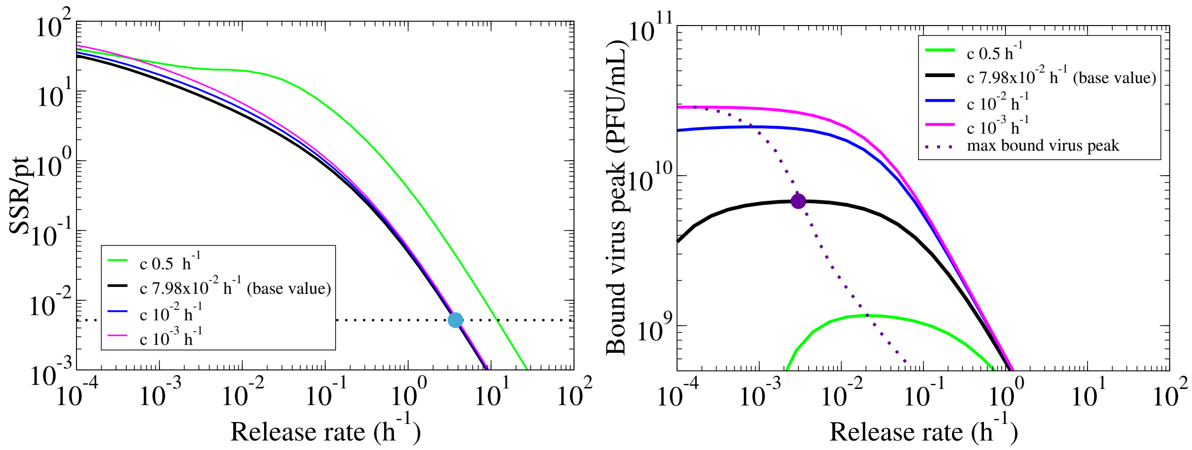

Fig S1. Determination of critical free and bound virus release rates, r_f and r_b. (Left) The sum-of-squared residuals per point (SSR/pt) is computed between each free virus curve in the release MM, as the release rate is varied, and the simulated MC data in the simple MM (black). The critical free virus release rate, $r_f = 3.72\ \mathrm{h}^{-1}$ (teal circle), corresponds to the variance of a mock-yield infection (dotted line). We also show the SSR/pt curve for various rates of loss of infectious virion that were explored (various colours). (Right) The peak value of bound virus titer as a function of the release rate is shown (black), where the maximum determines the critical bound virus release rate, $r_b = 3 \times 10^{-3}\ \mathrm{h}^{-1}$ (purple circle). The various coloured lines correspond to various rates of loss of virion infectivity to show that r_b (dotted purple) strongly depends on other infection parameters.

To verify whether r_f was an absolute value, or if it depended on the infection parameters, the parameters were individually varied away from their base values, and the SSR/pt curve was constructed again. In Fig S1 (left) we illustrate this procedure as the rate of loss of infectious virion, c, is varied. Lowering c to $10^{-3}\ \mathrm{h}^{-1}$ shifted the SSR/pt curve to the left, indicating a lower r_f. In Fig S2, we show that r_f only varied between $3\ \mathrm{h}^{-1}$–$5\ \mathrm{h}^{-1}$ for biologically reasonable values of the rate of loss of virion infectivity ($c < 0.2\ \mathrm{h}^{-1}$). We repeated this procedure for the remaining parameters and found that r_f weakly depends on the virus production rate, infection rate, eclipse length and infectious lifespan (Fig S2). Generally, however, r_f remains within the narrow range quoted.

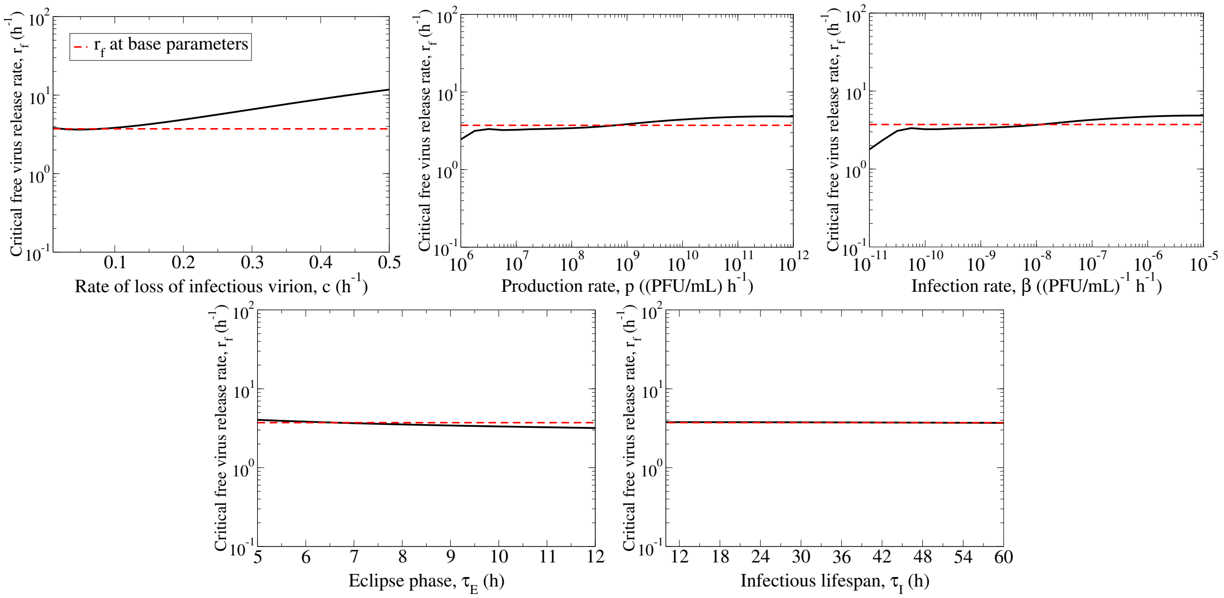

Fig S2. The critical free virus release rate, r_f, weakly depends on infection parameters. The critical free virus release rate, r_f, weakly depends on the rate of loss of virion infectivity, production rate, infection rate, eclipse phase and infectious lifespan. The r_f when all parameters are at their base values is indicated with a horizontal dotted line.

To quantitatively determine the critical bound virus release rate, Fig S1 (right) plots the peak value of bound virus as a function of the release rate. The maximum of the bound virus peak values determines r_b, indicating whether bound virus loses infectivity faster than it can be released ($r < r_b$), or vice versa ($r > r_b$). When all other parameters were at their base values, r_b was approximately 3×10^{-3} h^{-1}. Again, parameters were varied from their base values to determine their influence on r_b. In Fig S3, r_b was dependent on the rate of loss of virion infectivity, production rate, infection rate, and the infectious lifespan.

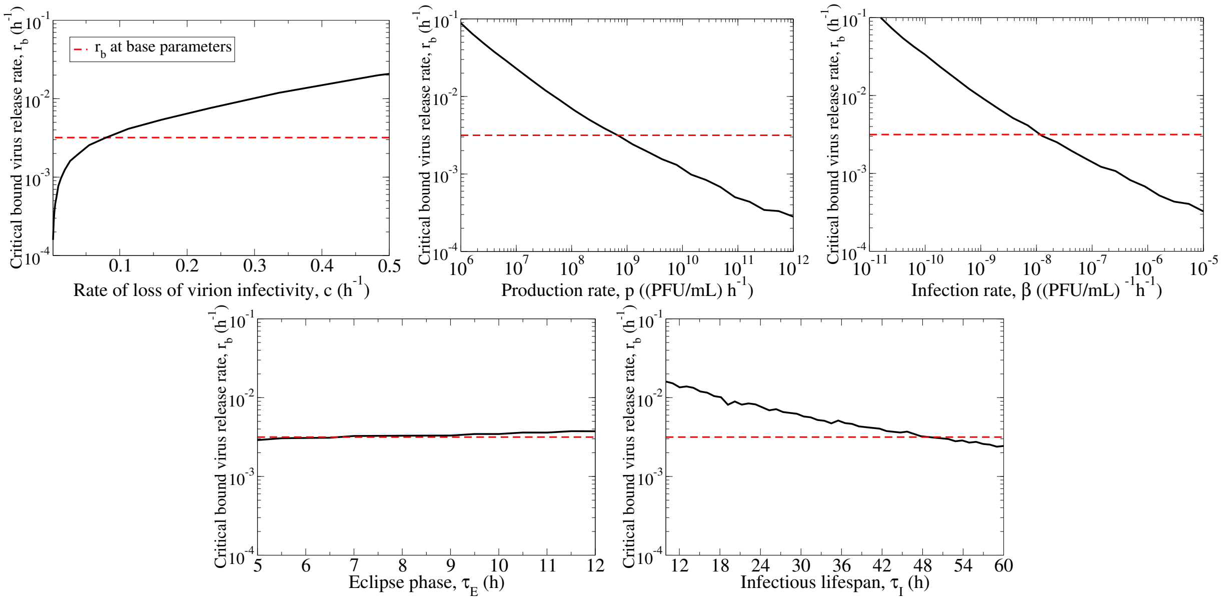

Fig S3. The critical bound virus release rate, r_b, depends on infection parameters. The critical bound virus release rate, r_b, depends on the rate of loss of virion infectivity, production rate, infection rate, eclipse phase, and infectious lifespan. The r_b when all parameters are at their base values is indicated with a black circle.

Infection parameters predicted by the release MM

When the release MM was fitted to simulated MC, SC, MY data from the simple MM, the simple MM did not significantly misestimate the infection rate or rate of loss of virion infectivity, as shown in Fig S4. We also examined a derived parameter, the basic reproductive number, R_0. The R_0 is defined as the number of secondary infections caused by a single infectious cell in a population of fully susceptible cells, and combines the influence of all the estimated infection parameters except for the eclipse length (Eq (8)). Fig S4 shows that the simple MM's estimate of R_0 does not significantly differ from the predicted value in the release MM.

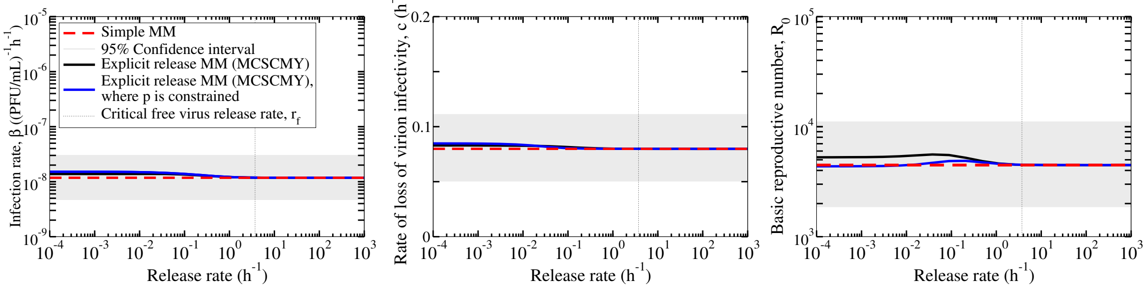

Fig S4. The simple MM does not significantly misestimate the infection rate, rate of loss of virion infectivity, or the basic reproductive number. As in Fig 4, but showing the infection rate, rate of loss of virion infectivity, and the basic reproductive number.

References

[1] Pinilla LT, Holder BP, Abed Y, Boivin G, Beauchemin CAA. The H275Y neuraminidase mutation of the pandemic A/H1N1 virus lengthens the eclipse phase and reduces viral output of infected cells, potentially compromising fitness in ferrets. J Virol. 2012;86(19):10651–10660. doi:10.1128/JVI.07244-11.
