# Supplementary figures and images for: Exploring virus release as a bottleneck for the spread of influenza A virus infection in vitro and the implications for antiviral therapy with neuraminidase inhibitors
Source: PLoS One. 2017 Aug 24;12(8):e0183621. doi: 10.1371/journal.pone.0183621 (PMC5570347; doi:10.1371/journal.pone.0183621)

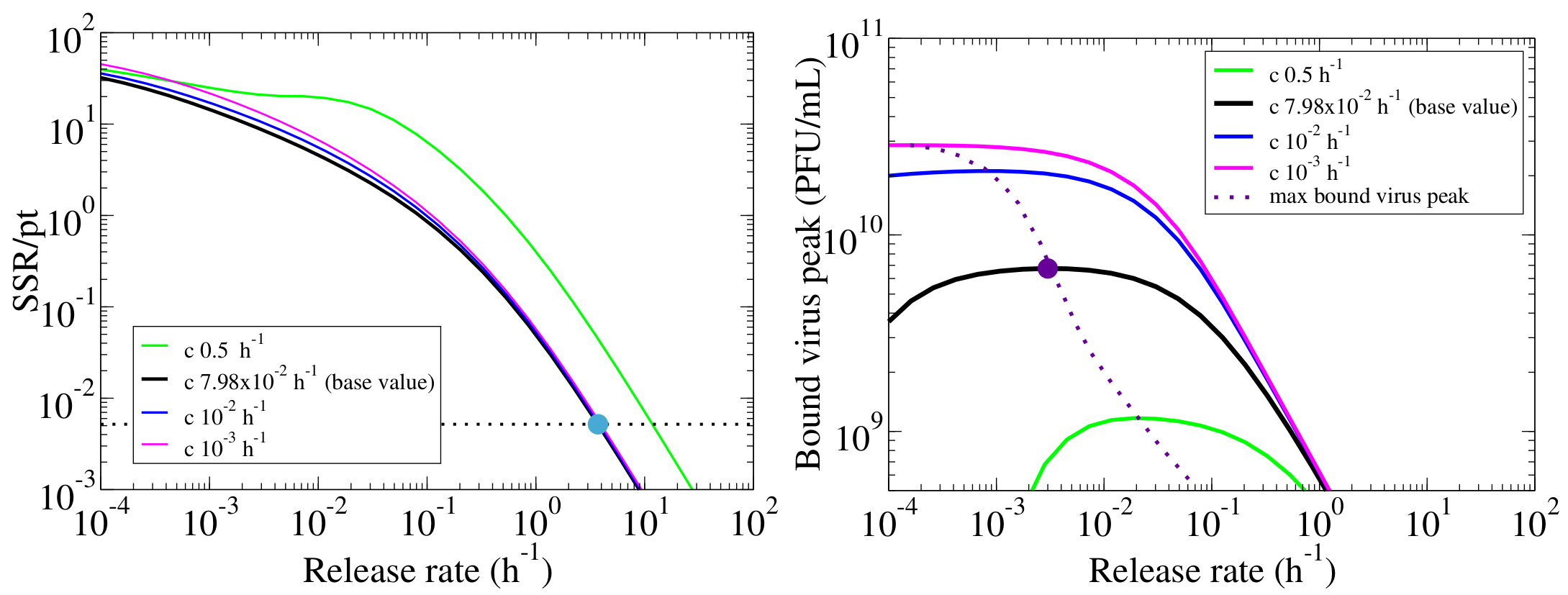

Supplement: S1 Fig — (Left) The sum-of-squared residuals per point (SSR/pt) is computed between each free virus curve in the release MM, as the release rate is varied, and the simulated MC data in the simple MM (black). The critical free virus release rate, rf = 3.72 h−1 (teal circle), corresponds to the variance of a mock-yield infection (dotted line). We also show the SSR/pt curve for various rates of loss of infectious virion that were explored (various colours). (Right) The peak value of bound virus titer as a function of the release rate is shown (black), where the maximum determines the critical bound virus release rate, rb = 3 × 10−3 h−1 (purple circle). The various coloured lines correspond to various rates of loss of virion infectivity to show that rb (dotted purple) strongly depends on other infection parameters. (TIF) [file pone.0183621.s002.tif]

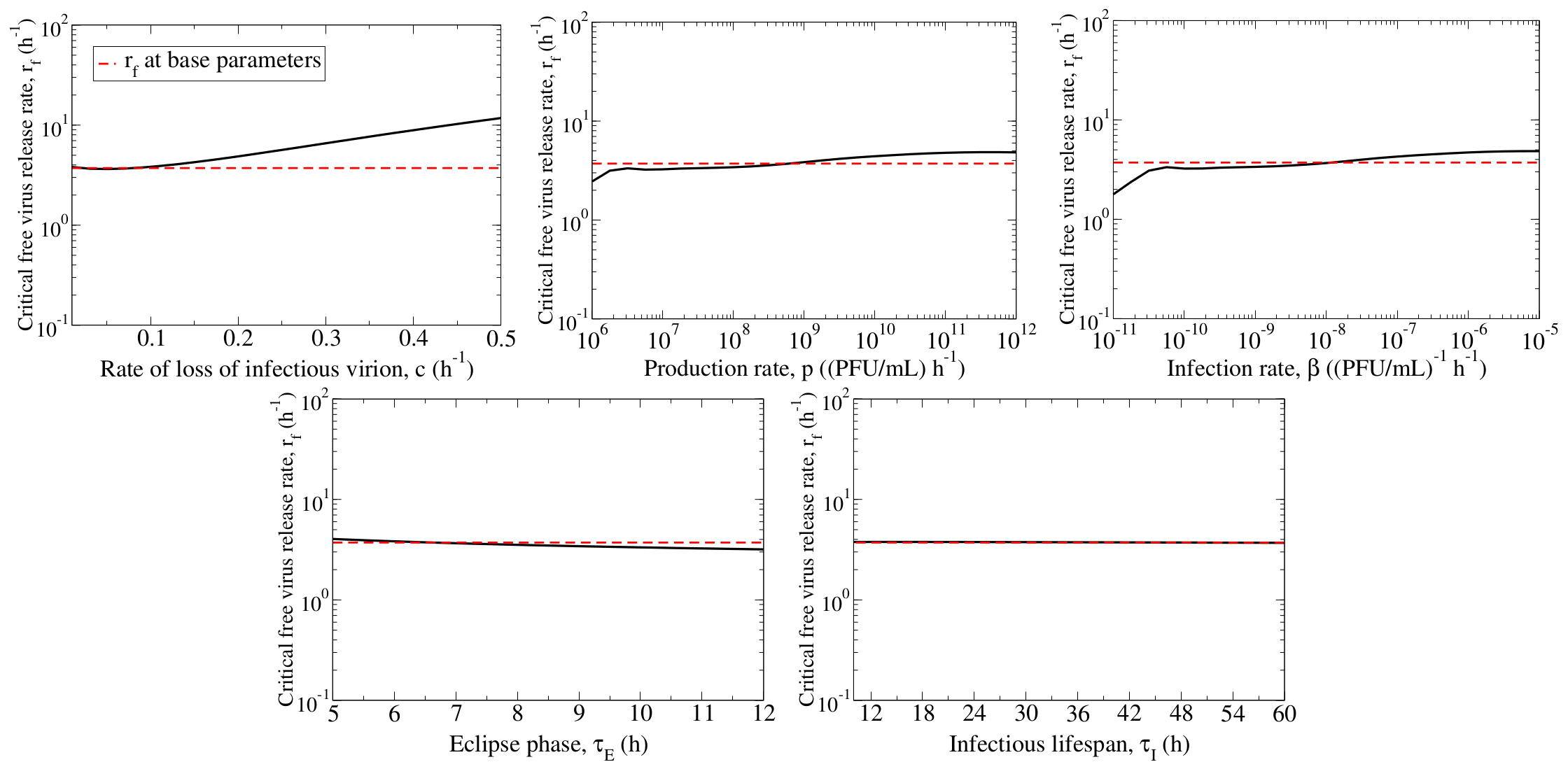

Supplement: S2 Fig — The critical free virus release rate, rf, weakly depends on the rate of loss of virion infectivity, production rate, infection rate, eclipse phase and infectious lifespan. The rf when all parameters are at their base values is indicated with a horizontal dotted line. (TIF) [file pone.0183621.s003.tif]

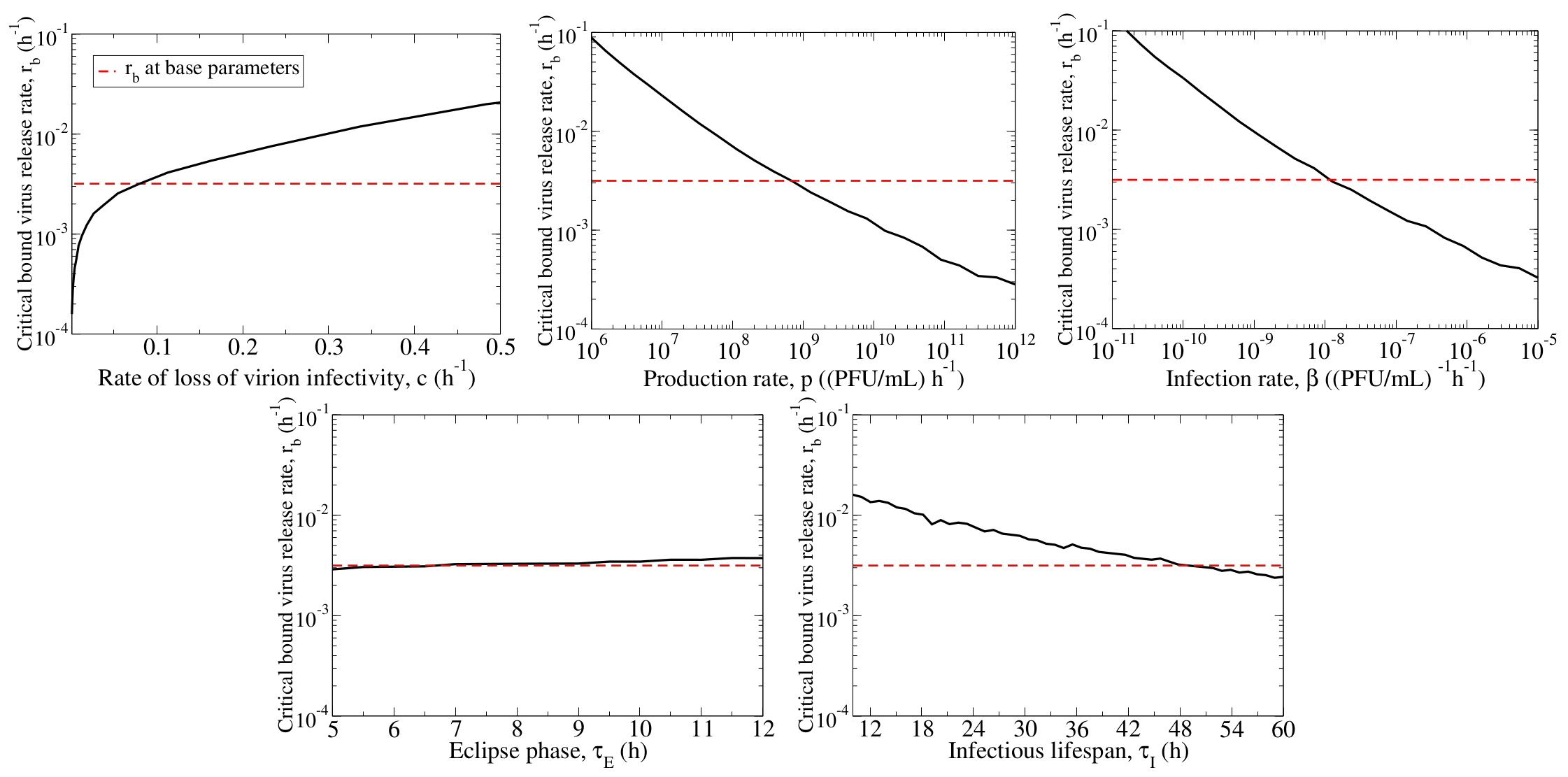

Supplement: S3 Fig — The critical bound virus release rate, rb, depends on the rate of loss of virion infectivity, production rate, infection rate, eclipse phase, and infectious lifespan. The rb when all parameters are at their base values is indicated with a black circle. (TIF) [file pone.0183621.s004.tif]

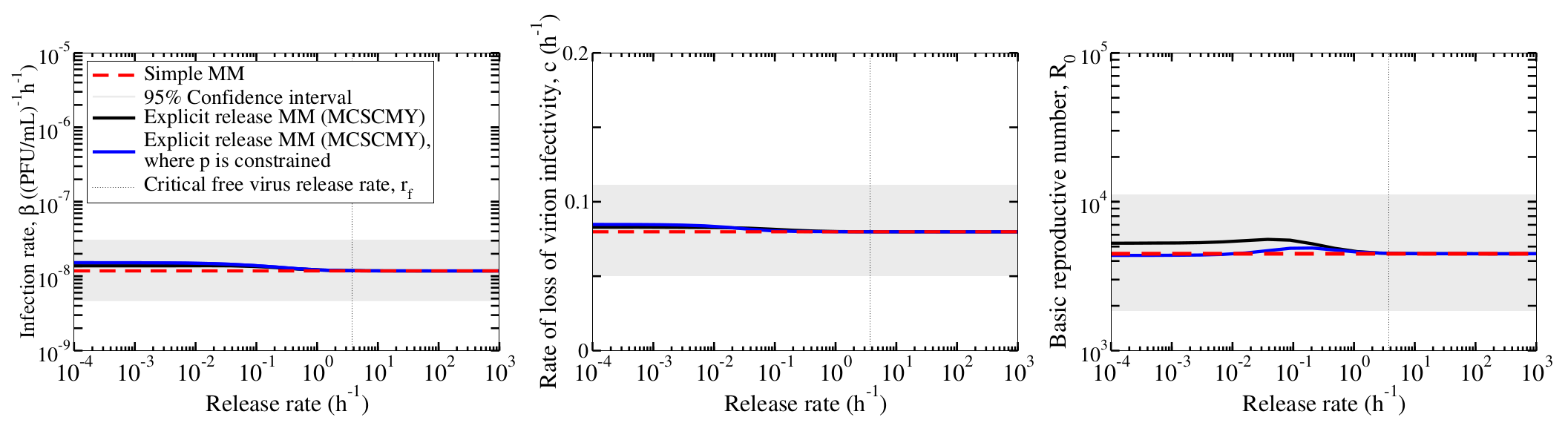

Supplement: S4 Fig — As in Fig 4, but showing the infection rate, rate of loss of virion infectivity, and the basic reproductive number. (TIF) [file pone.0183621.s005.tif]
